# Acetylcholine-treated murine dendritic cells promote inflammatory lung injury

**DOI:** 10.1371/journal.pone.0212911

**Published:** 2019-03-01

**Authors:** Soledad Gori, Julieta Alcain, Silvia Vanzulli, Mariela A. Moreno Ayala, Marianela Candolfi, Carolina Jancic, Jorge Geffner, Mónica Vermeulen, Gabriela Salamone

**Affiliations:** 1 Instituto de Medicina Experimental (IMEX), CONICET, Academia Nacional de Medicina, Buenos Aires, Argentina; 2 Laboratorio de Anatomía Patológica, Instituto de Estudios Oncológicos, Academia Nacional de Medicina, Buenos Aires, Argentina; 3 Instituto de Investigaciones Biomédicas (INBIOMED UBA-CONICET), Facultad de Medicina, Universidad de Buenos Aires, Buenos Aires, Argentina; 4 Departamento de Microbiología, Parasitología e Inmunología, Facultad de Medicina, Universidad de Buenos Aires, Buenos Aires, Argentina; 5 Instituto de Investigaciones Biomédicas en Retrovirus y SIDA (INBIRS), CONICET, Facultad de Medicina, Universidad de Buenos Aires, Buenos Aires, Argentina; Centre National de la Recherche Scientifique, FRANCE

## Abstract

In recent years a non-neuronal cholinergic system has been described in immune cells, which is often usually activated during the course of inflammatory processes. To date, it is known that Acetylcholine (ACh), a neurotransmitter extensively expressed in the airways, not only induces bronchoconstriction, but also promotes a set of changes usually associated with the induction of allergic/Th2 responses. We have previously demonstrated that ACh polarizes human dendritic cells (DC) toward a Th2-promoting profile through the activation of muscarinic acetylcholine receptors (mAChR). Here, we showed that ACh promotes the acquisition of an inflammatory profile by murine DC, with the increased MHC II IA^d^ expression and production of two cytokines strongly associated with inflammatory infiltrate and tissue damage, namely TNF-α and MCP-1, which was prevented by blocking mAChR. Moreover, we showed that ACh induces the up-regulation of M3 mAChR expression and the blocking of this receptor with tiotropium bromide prevents the increase of MHC II IAd expression and TNF-α production induced by ACh on DC, suggesting that M3 is the main receptor involved in ACh-induced activation of DC. Then, using a short-term experimental murine model of ovalbumin-induced lung inflammation, we revealed that the intranasal administration of ACh-treated DC, at early stages of the inflammatory response, might be able to exacerbate the recruitment of inflammatory mononuclear cells, promoting profound structural changes in the lung parenchyma characteristic of chronic inflammation and evidenced by elevated systemic levels of inflammatory marker, TNF-α. These results suggest a potential role for ACh in the modulation of immune mechanisms underlying pulmonary inflammatory processes.

## Introduction

Because the lung is an organ that is largely exposed to microenvironmental stimuli such as microbes, allergens and pollution, the local presence of antigen-presenting cells, such as dendritic cells (DC), is crucial for the initiation and maintenance of mucosal immunity in the respiratory tract. It is known that stimulated DC are essential for resting T cell activation and their differentiation into distinct functional profiles. Besides pathogen-associated molecular patterns and cytokines, other stimuli are capable to modulate DC function [1].

In recent years it has become evident there is interaction between the nervous and immune systems [2–4]. A non-neuronal cholinergic system has been described in immune cells, which is usually activated during the course of inflammatory processes [5–7]. Acetylcholine (ACh) represents the most important neurotransmitter of the parasympathetic nervous system. This molecule is largely expressed in the airways, where it induces smooth muscle contraction [5,8]. For this reason, anticholinergic drugs are commonly used as bronchodilators in obstructive airway diseases. Interestingly, recent studies revealed that ACh not only induces bronchoconstriction, but also promotes eosinophilia, mucus overproduction and airway remodeling, a set of changes usually associated with the induction of allergic/Th2 responses [8]. Moreover, we have recently demonstrated that ACh polarizes DC toward a Th2-promoting profile through the activation of muscarinic acetylcholine receptors (mAChR) [9]. In addition, Bühling *et al* have shown that ACh-induced release of chemotactic mediators from inflammatory cells could be inhibited by tiotropium bromide (Tio), a selective M3 mAChR antagonist [10].

Here, we found that ACh supports a pro-inflammatory response acting on DC. Using a short-term model of ovalbumin (OVA)-induced lung inflammation, we revealed that the intranasal (i.n.) administration of ACh-treated DC might be able to exacerbate this inflammatory response, increasing lung injury by the induction of mononuclear cell recruitment.

## Methods

### 2.1. Mice

All experiments were carried out using 2-month-old virgin female BALB/c or C57BL/6 mice raised at the Instituto de Medicina Experimental (IMEX), CONICET, Academia Nacional de Medicina, Buenos Aires, Argentina. They were housed six per cage and kept at 20° ± 2° under an automatic 12 h light–dark schedule, according to the National Institute of Health guidelines. All experimental protocols used in this research were approved by Institutional Animal Care and Use Committee of IMEX, according to American Physiological Society’s Guiding Principles for the Care and Use of Animals in Research and following local normative regulations.

### 2. 2. DC generation from bone marrow cultures

DC were differentiated from BALB/c bone marrow precursors, as described by Inaba *et al* [11], with minor modifications reported previously [12]. Briefly, the precursors were cultured at 1 x 10^6^ cells/ml in RPMI-1640 complete medium, supplemented with 30% conditioned medium from GM-CSF-producing J558 cells. Upon differentiation, DC were incubated (1 x 10^6^ cells/ml) with or without different concentrations of ACh (Sigma Aldrich, St. Louis, MO, USA) for 18 h, and phenotypic changes were analyzed. In some experiments, DC were pre-incubated for 30 min with or without the nonselective mAChR and nicotinic (nAChR) antagonists, atropine (AT 10^-9^M, Laboratorio Larjan, Bs. As., Argentina) and mecamylamine (MM 10^-9^M, Sigma Aldrich), respectively. In some cases, DC were pre-incubated with a selective M3 mAChR antagonist, Tio (30nM, Boehringer Ingelheim). DC treated with lipopolysaccharide or LPS (from *E*. *coli* 0111:B4, Sigma Aldrich) 1 μg/ml for 18h were used as positive control of cell activation. Cell viability of cultures was evaluated by trypan blue exclusion dye assay.

### 2.3. Mixed leukocyte reaction (MLR)

DC (5 x 10^4^ cells) differentiated from bone marrow precursors obtained from BALB/c mice were treated or untreated with ACh (ACh-DC or DC; respectively), then washed and co-cultured with splenocytes from C57BL/6 mice (1:5 ratio) for 5 days. Proliferation was determined by thymidine incorporation measured as described in [9].

### 2. 4. Sensitization and challenge of mice with OVA

**Fig 1:**
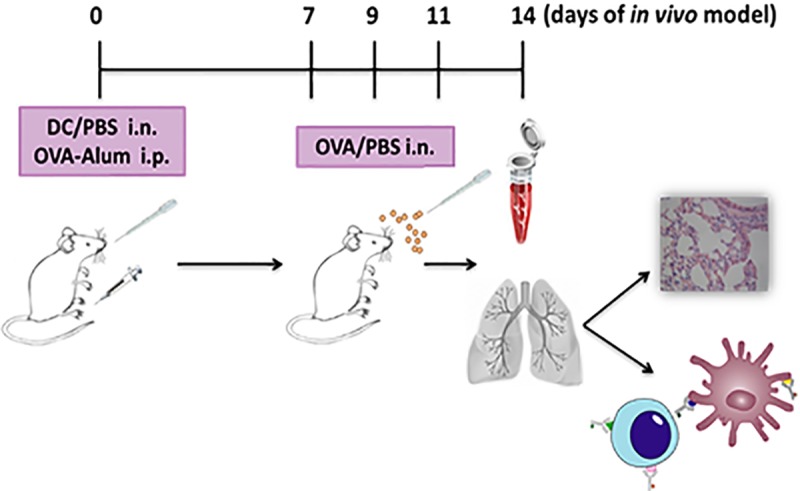
Schematic experimental design of OVA-induced lung inflammation murine model. Our experiments involved the use of 4 different mice groups (4 mice/group), as described in Methods. In all cases, mice were sacrificed at day 14 and the lungs were isolated for analysis.

An experimental model of ovalbumin (OVA)-induced lung inflammation was performed in BALB/c mice as previously described [12–14], with minor modifications (Fig 1). Four experimental mice groups were used in this study. Briefly, at day 0, DC untreated or treated with ACh 10^-11^M for 18 h were washed and transferred via the i.n. route (5x10^4^ DC/100 μl PBS) to groups I and II, respectively. In parallel, mice in groups III and IV received PBS (100 μl PBS), instead of DC. Ten minutes after i.n. instillation of DC (groups I and II) or PBS (groups III and IV), all groups received OVA-sodium aluminate (Alum) solution (50 μg OVA and 1 mg Alum/500 μl PBS, Sigma Aldrich) intraperitoneally (i.p.). Finally, on days 7, 9, and 11, groups I, II and III were challenged with OVA (0.1 μg/50 μl PBS) via the i.n. route, while group IV (control) received PBS instead of OVA. All groups were sacrificed on day 14 by cervical dislocation resulting in euthanasia within 10 seconds and serum samples were obtained as previously described in [12].

### 2.5. Lung pathology

The isolated lungs were inflated with 4% paraformaldehyde-PBS prior to immersion fixation for 24 h and paraffin-embedded. Four-micron sections were stained with haematoxylin and eosin (H&E) and periodic acid-Schiff (PAS). Representative microphotographs were obtained with a Zeiss Axiolab microscope. For peribronchial mononuclear infiltrate quantification, inflammatory cells were counted at x40 magnification in at least ten fields on sections stained with H&E. Results were expressed as fold increase. Alveolar involvement was evaluated according to a scoring system adapted from Bayes *et al* 2016 [15] (Table 1). Lung sections stained with PAS were examined at x10 magnification in both central and peripheral airways with epithelial layer intact for evaluate the area proportion of mucus. The difference between luminal area with and without mucus was determined using microscope software platform Leica Application Suite (LAS4). Results were expressed as % and the mean ± SEM was calculated for each experimental group.

**Table 1:** Histological scoring system for inflammation in lungs of mice treated with DC and ACh-DC.

Score	Alveolar Involvement
0	None
1	Mild (patchy increased cellularity without septal thickening)
2	Moderate (increased cellularity with septal thickening)
3	Severe (25–50% visualized lung with increased cellularity/thickening)
4	Diffuse (>50% visualized lung with increased cellularity/thickening)

Randomly, selected sections were scored blindly according to the review of a complete lung section, using an x10 magnification. Adapted from Bayes et al 2016 [15].

### 2.6. Treatment of lungs to obtain cell suspension

Lungs were cut into small pieces and treated with Type I collagenase and DNase I, as described previously [12]. The cell suspensions were collected through a gauze mesh and washed with cold PBS.

### 2.7. Evaluation of serum levels of IgE antibodies directed to ovalbumin

Ovalbumin-specific IgE antibody levels were determined from serum samples using a rat anti-mouse IgE (BD Biosciences) as described previously in [12].

### 2.8. Cytokines determination by ELISA

Cytokines were evaluated using commercial kits: TNF-α, MCP-1 (e-Biosciences, San Diego, CA, USA), IL-12p70 and IL-10 (BD Biosciences), according to the manufacturer’s recommendations.

### 2.9. Flow cytometry

Cell staining was performed using FITC- or PE-conjugated monoclonal antibodies directed to CD11c, MHC II IA^d^, Gr-1, CD86, CD11b, CD4, CD8, B220 or the corresponding isotype control (BD Biosciences), according to the manufacturer’s recommendations. The expression of M3 mAChR was evaluated using specific goat IgG polyclonal antibody (Santa Cruz Biotechnology, Germany) and secondary Alexa 488-labeled polyclonal IgG antibody directed to goat IgG (Sigma Aldrich) as previously described in [9]. The data were collected using a BD FACSCalibur flow cytometer (BD Biosciences) and analysis was performed using the FlowJo 7.6 software.

### 2. 10. Statistical analysis

Statistical significance was determined using the nonparametric Friedman test or Kruskal–Wallis test with Dunn’s post-test analysis for multiple comparisons and Wilcoxon test for simple comparisons. Statistical significance was defined as p<0.05 with GraphPad Prism 6 software (San Diego, CA, USA).

## Results and discussion

**Fig 2:**
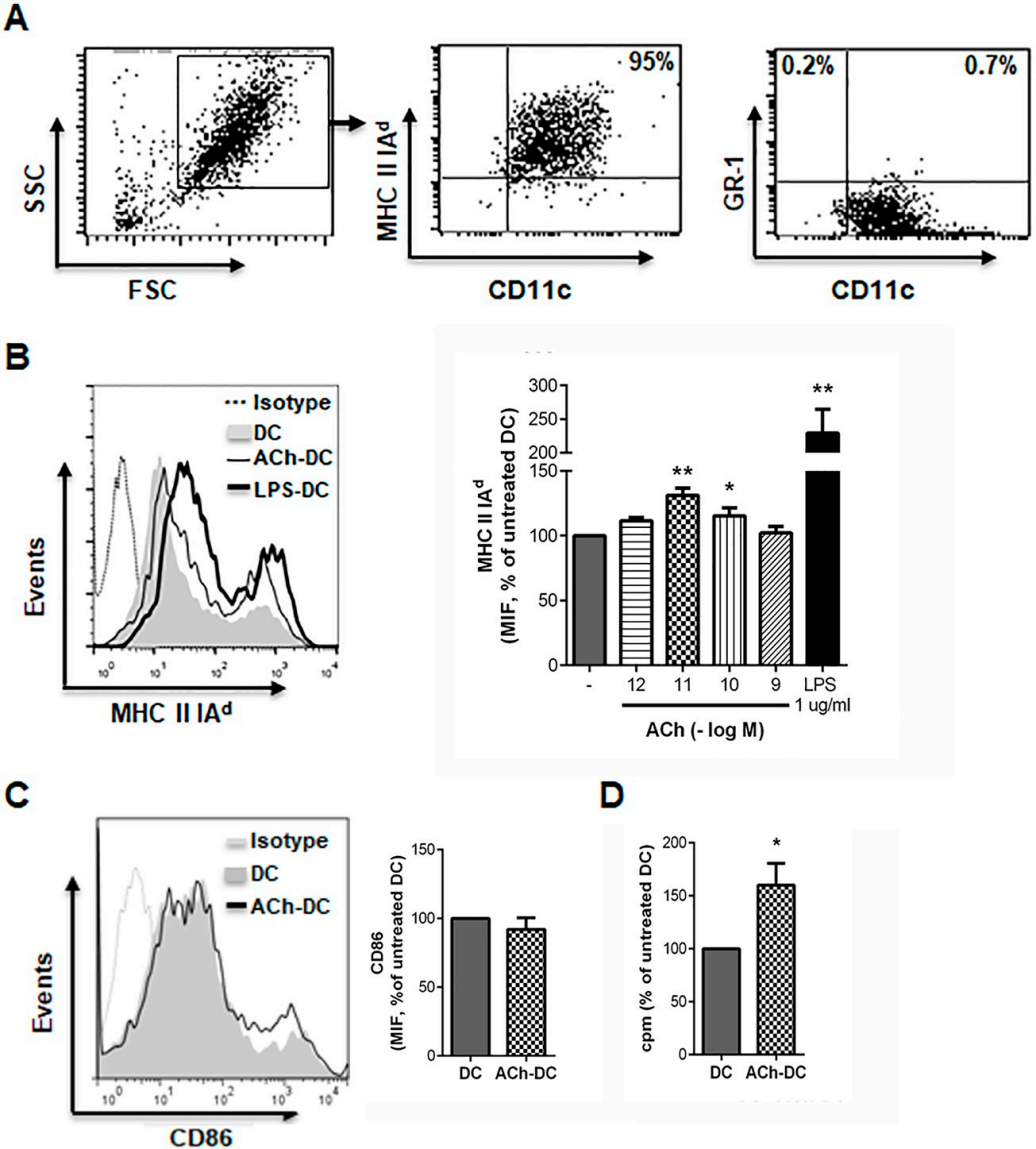
ACh increases the expression of MHC class II and the stimulatory ability of murine DC. (A) Phenotype of immature DC differentiated from bone marrow precursors from BALB/c mice was analyzed by flow cytometry. A representative experiment is shown. (B-C) DC (1x10^6^ cells/ml) were treated or not with different concentrations of ACh or LPS 1μg/ml for 18h and the expression of MHC class II (anti-IA^d^) was evaluated by flow cytometry (B), while CD86 expression (C) was evaluated on ACh 10^−11^ M-treated DC. Representative histograms of median intensity of fluorescence (MIF) are shown and bars represent the mean ± SEM of 5–7 experiments (2 replicates/experiment). *p<0.05, **p<0.01 *vs* untreated DC, nonparametric Friedman test. (D) ACh 10^−11^ M-treated-DC and DC (5x10^4^ cells) were washed and co-cultured with C57BL/6 splenocytes (1:5 ratio) for 5 days. The proliferation was determined by thymidine incorporation and data are expressed as the mean ± SEM of ^[3H]^ thymidine incorporation (n = 5 experiments, 2 replicates/experiment). *p<0.05, nonparametric Wilcoxon test.

We aimed to evaluate whether ACh acting on DC affects the early development of the inflammatory response in the lung, and based on our previous results in human DC [9,16], we first assessed whether this neurotransmitter is able to modulate the phenotypic and functional profile of murine DC. For this, we differentiated DC from bone marrow precursors and at day 9 of culture, we corroborated that over 85% of the harvested cells were MHC class II IA^d^+/CD11c+ and that the contamination with granulocytes (GR-1+CD11c- cells) was less than 1%, as shown in Fig 2A. Next, DC were cultured for 18 h with or without different concentrations of ACh and DC treated with ACh 10^-11^M showed the higher increase of the expression of MHC class II IA^d^, as shown in Fig 2B (a positive control of DC activation with LPS is shown), while CD86 expression was not affected (Fig 2C). As shown in Fig 2D, the treatment of DC with ACh resulted in enhanced proliferation of C57BL/6 splenocytes in MLR.

**Fig 3:**
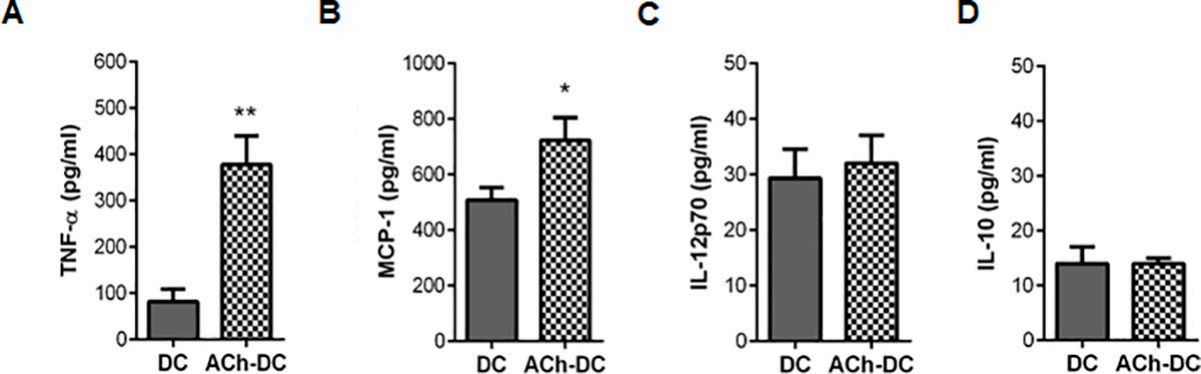
ACh enhances the production of pro-inflammatory cytokines on murine DC. (A-D) DC (1x10^6^ cells/ml) were incubated with or without ACh 10^−11^ M for 18 h. TNF-α (A), MCP-1 (B), IL-12p70 (C) and IL-10 (D) secretion was measured by ELISA (mean ± SEM, n = 4 experiments). *p<0.05; **p<0.01 nonparametric Wilcoxon test.

Then, similarly to described in human DC [9], we found that ACh markedly stimulated the production of TNF-α (a positive control with LPS is shown in S1 Fig) without modifying the spontaneous production of IL-12p70 and IL-10 (Fig 3C and 3D). Surprisingly, we found that ACh also significantly increased the secretion of CCL2/MCP-1 chemokine (Fig 3B).

**Fig 4:**
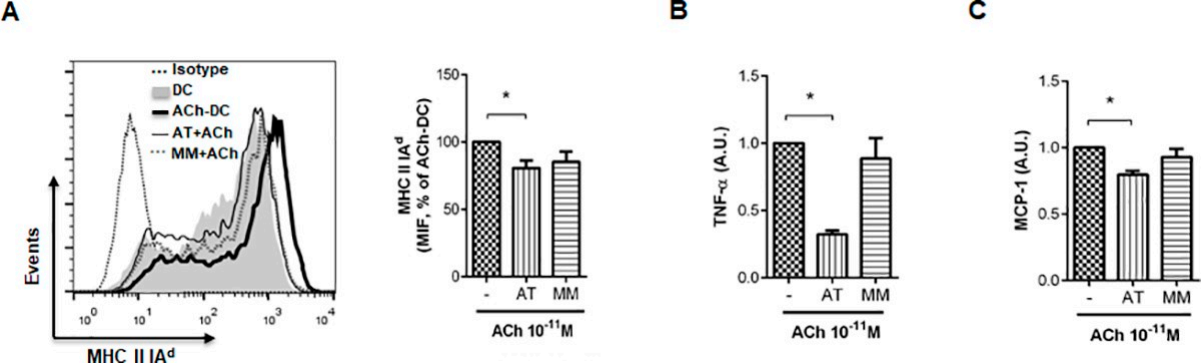
ACh-induced activation of DC is mediated by mAChR. (A-C) DC (1x10^6^ cells/ml) were exposed or not to cholinergic receptors antagonists (AT and MM, 10^-9^M for 30 m) and then were incubated with or without ACh 10^−11^ M for 18 h. MHC Class II IA^d^ expression (A) was measured by flow cytometry and a representative histogram of MIF of n = 6 experiments is shown (mean ± SEM, 2 replicates/experiment). (B-C) TNF-α (B) and MCP-1 (C) secretion was measured by ELISA (mean ± SEM, n = 4 experiments). *p<0.05, nonparametric Kruskal-Wallis for multiple comparisons with Dunn’s post-test. A. U.: arbitrary units.

Because DC express both m- and nAChR [6,7], we then analyzed which of these receptors was involved in the promotion of the observed effects. To this aim, we used nonselective m- and nAChR antagonists: AT and MM, respectively. Fig 4A–4C shows that AT, but not MM, significantly prevented the ACh-induced increment of MHC II IA^d^ expression and TNF-α and MCP-1 production, suggesting the involvement of mAChR in DC stimulation by ACh, as we previously observed in human DC [9].

In agreement with our results, several *in vitro* and *in vivo* studies support the fact that ACh, acting on mAChR, exerts a pro-inflammatory role on airway immune and nonimmune cells [17–19] and contributes to airway inflammation, Th2 cytokines production and remodeling in obstructive airway diseases, primarily via M3 receptor [8,20]. Also it has been shown that by blocking mAChR with nonselective (AT) or selective to M3 (Tio or 4-DAMP) antagonists, it is possible to prevent these effects observed *in vitro* and in asthma models [8,10,20,21]. Bühling *et al* [10] reported that ACh-stimulated alveolar macrophages, monocytes and bronchial epithelial cells enhanced the release of chemotactic active mediators which was prevented by Tio. On the contrary, this effect was not observed in lung fibroblasts, associated with the lower amount of M3 expression and a predominance of M2, a mAChR associated with inhibitory functions [22]. Furthermore, Profita *et al* [23] showed that M2 expression is significantly decreased in macrophages and neutrophils of COPD patients sputum, contrary to M3 expression.

**Fig 5:**
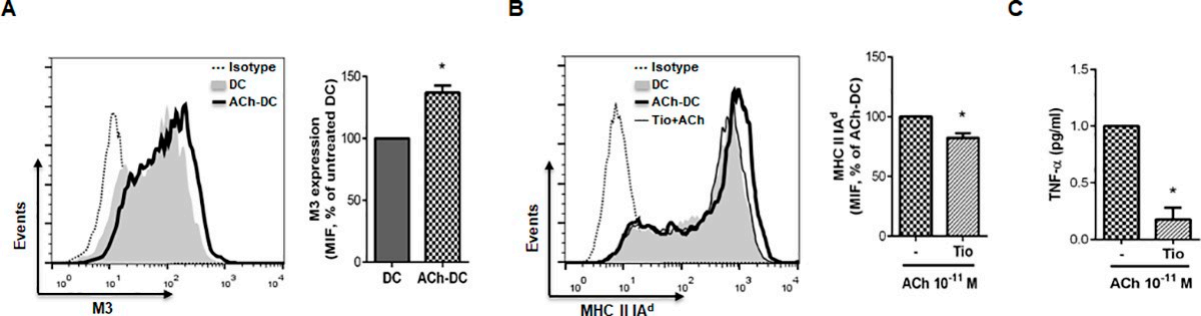
M3 mAChR is involved in ACh-induced activation of DC. (A-C) DC (1x10^6^ cells/ml) were exposed or not to selective M3 receptor antagonist (Tio 30nM for 30 m) and then were incubated with or without ACh 10^−11^ M for 18 h. M3 (A) and MHC II IA^d^ (B) expression was determined by flow cytometry and representative histograms of MIF of 5 experiments are shown (mean ± SEM, 2 replicates/experiment). TNF-α production (C) was measured by ELISA (mean ± SEM, n = 4 experiments). *p<0.05, nonparametric Wilcoxon test.

In line with these reports, we observed that M3 expression on bone-marrow DC is significantly up-regulated by ACh (Fig 5A). Moreover, when DC were pre-treated with Tio, the ACh-induced increase of MHC II IA^d^ expression and TNF-α production was significantly prevented (Fig 5B and 5C, respectively). Notably, pre-treatment with Tio reached similar blockade levels compared to pre-treatment with the nonselective mAChR antagonist, AT, suggesting that M3 might be the main receptor involved in ACh-induced activation of DC.

It is well established the use of Tio as a long-acting M3 muscarinic antagonist that exerts a bronchodilator effect on the airway, but taking the above-mentioned points into account, we infer that Tio could contribute to the decrease of the exacerbation rate observed in COPD and severe persistent asthma treated patients [24,25], acting also on inflammatory response.

In sharp contrast to the pro-inflammatory role of mAChR stimulation, nAChRs appear to play an important anti-inflammatory role in many cell types and organs. This immunosuppressive effect has been well established in DC, monocytes, macrophages, endothelial cells and neutrophils [26–32], with a decrease in the production of pro-inflammatory cytokines such as TNF-α [32].

Taken together, these results suggest that ACh modulates the phenotype and function of mouse and human DC in a similar fashion, inducing them to a pro-inflammatory profile mediated by mAChR signaling, mainly the M3 receptor.

Finally, we evaluated the impact of ACh-treated DC on the early inflammatory response, using a short-term experimental model of OVA-induced lung inflammation [13,14], with minor modifications illustrated in Fig 1.

**Fig 6:**
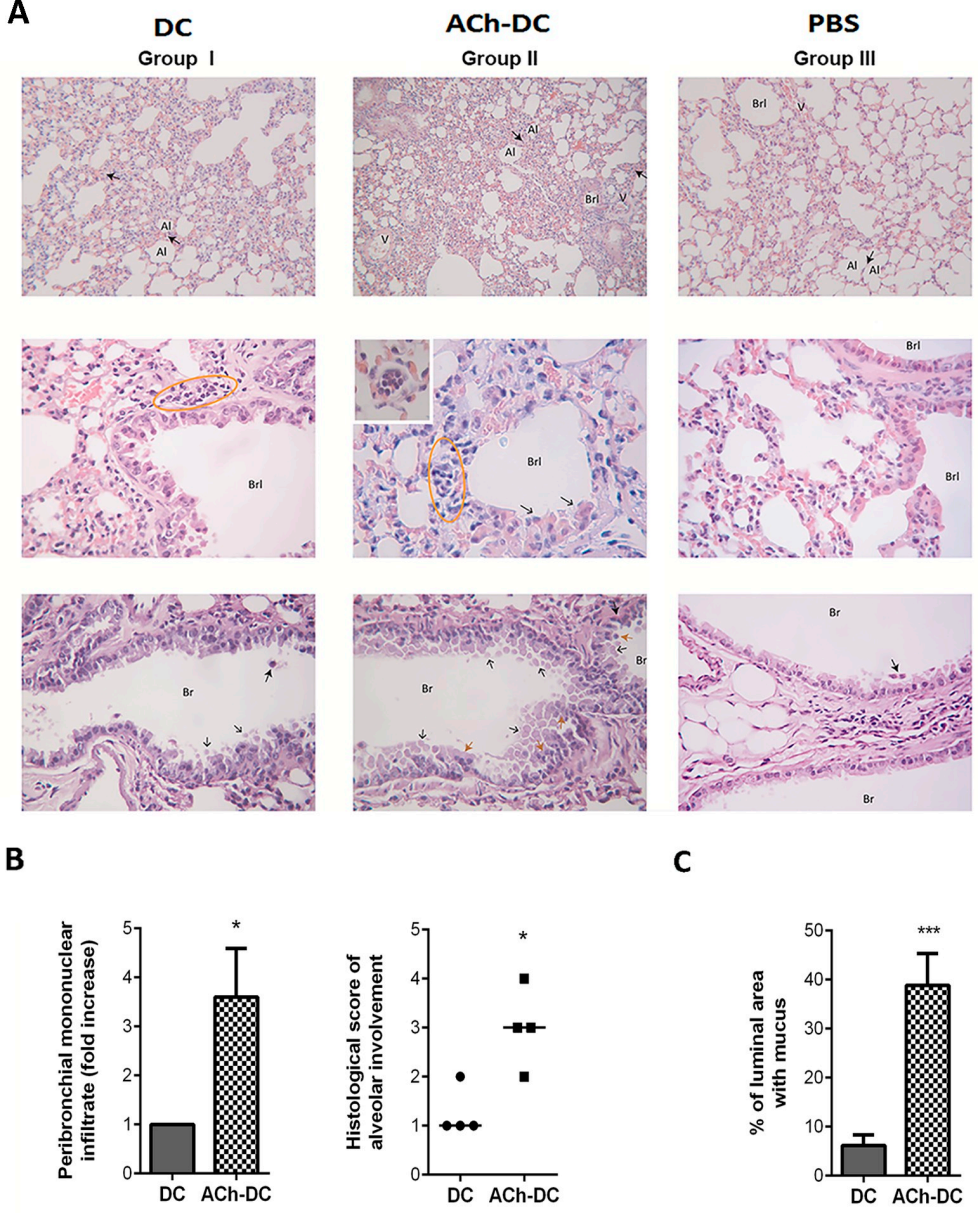
ACh-DC facilitates the development of inflammatory process in onset of OVA-induced lung inflammation murine model. (A) H&E (upper and middle panels) and PAS staining (lower panel) of fixed lungs from mice of groups I-III are shown. In the upper panel, the full arrows indicate the alveolar septum (x50 magnification). In the middle panel the ellipses indicate peribronchiolar mononuclear infiltrate, the black arrows indicate epithelial damage and the insert in group II shows the recruitment of mononuclear cells into blood vessel (x400 magnification). In the lower panel, the full arrows indicate PAS positive cells, the black arrows indicate mucin secretion while the orange arrows indicate epithelial desquamation (x400 magnification). Al: Alveolus, V: Blood vessel, Br: Bronchus, Brl: Bronchiole. A representative experiment is shown (n = 4). (B) Peribronchial mononuclear infiltrate was quantified at x40 magnification in at least ten fields on sections stained with H&E and results are expressed as mean ± SEM of fold increase (B, left panel) while the alveolar involvement was evaluated according to a scoring system described in Methods (B, right panel). (C) Lung sections stained with PAS were examined at x10 magnification and the area proportion of mucus in % (mean ± SEM) was determinate as described in Methods. *p<0.05; ***p<0.001 nonparametric Wilcoxon test, n = 4 experiments.

Interestingly, the histopathological analysis of lung from OVA-challenged mice revealed that mononuclear cell infiltration was higher in the lungs from mice instilled with ACh-DC (group II) compared with mice instilled with untreated-DC (group I), while a low infiltration pattern was observed in mice instilled with PBS (group III) (Fig 6A, upper panel). Moreover, the massive infiltration of mononuclear cells observed in group II was shown to be associated to septal thickening and decreased lumen with loss of distal air space and normal structure of bronchioles, due to epithelial damage. These changes were less pronounced in mice receiving untreated DC (Fig 6A, upper and middle panels). Cell desquamation, epithelial cell hyperplasia and metaplasia of mucus secreting cells, as well as the amount of secreted mucus, was higher in lungs from group II compared with those treated with group I (Fig 6A, lower panel). The increase in peribronchial mononuclear infiltration, alveolar involvement and mucus production observed in group II was significant compared to group I, as shown in Fig 6B and 6C.

**Fig 7:**
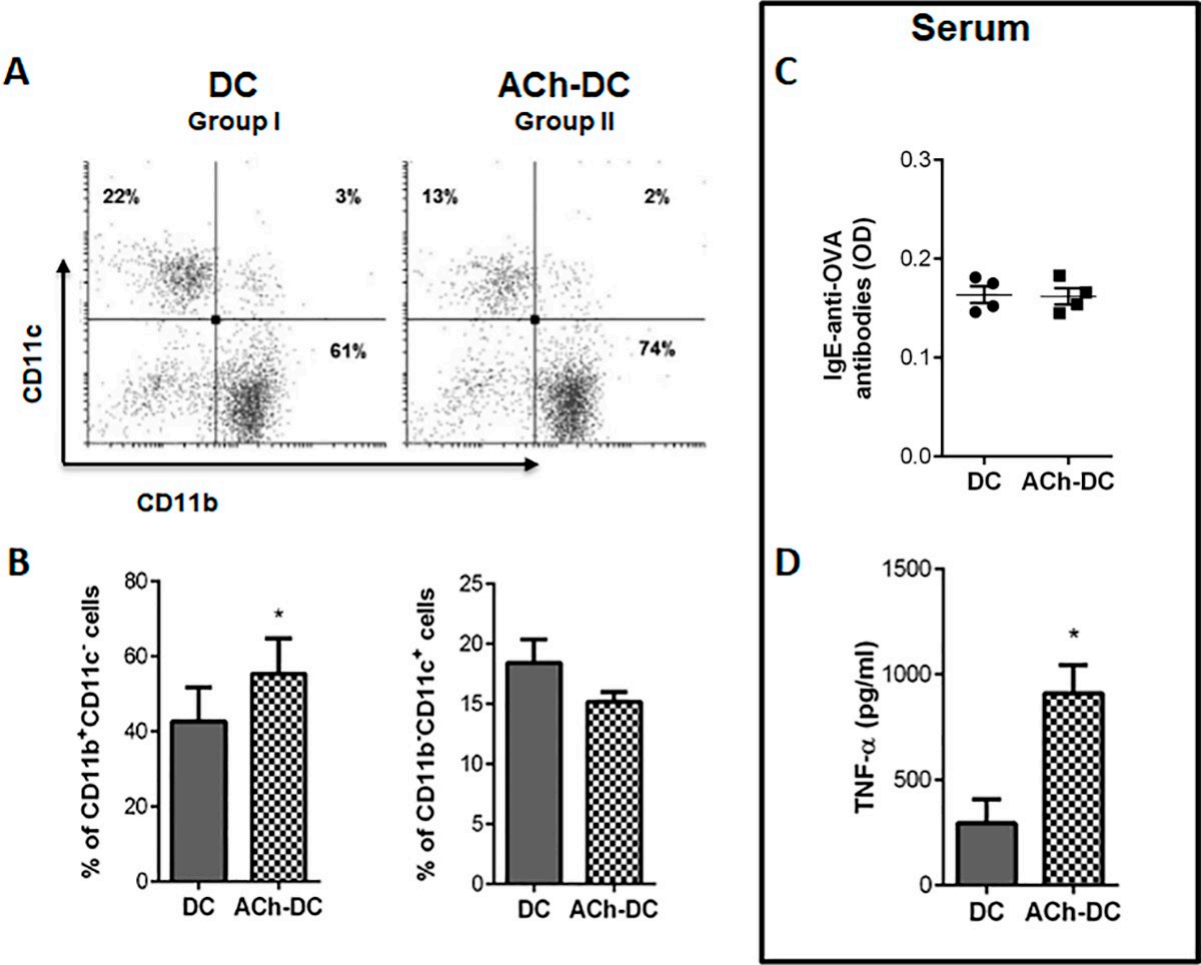
ACh-DC induces the recruitment of CD11b^+^CD11c^-^ cells in the onset of OVA-induced lung inflammation accompanied by increased TNF-α production. (A-B) Cells isolated from lungs were stained with mAb directed to CD11c and CD11b and analyzed by flow cytometry, gating mononuclear cells and excluding for the analysis the High Autofluorescence Cells. A representative experiment (A) and the quantification of CD11b^+^CD11c^-^ and CD11b^-^CD11c^+^ cells (B) are shown (mean % ± SEM, n = 4 experiments). (C-D) Level of circulating anti-OVA IgE antibodies (C) and TNF-α (D) was measured at the end of the *in vivo* model in mice serum samples of day 14 by ELISA (mean ± SEM, n = 4 experiments). (C) A representative experiment is shown (OD media ± SEM, n = 4 animals/group). *p<0.05 nonparametric Wilcoxon test.

Furthermore, we observed a significant increase in the population of CD11b^+^CD11c^-^ cells in lungs of group II respect to the group I (Fig 7A and 7B). No differences were observed in CD4^+^, CD8^+^ or B220^+^ cells as shown in S2 Fig. These results were consistent with those of Gonzalo *et al* [14,33] which showed that at day 15 of an OVA-induced lung inflammation model, the main population of lung-infiltrating immune cells was monocytic/macrophagic while the lymphocytic was the lowest. Related to this, we observed a similarly low level of circulating anti-OVA IgE antibodies at the end of the *in vivo* model in all of OVA-instillated groups (Fig 7C), accordingly with reports of Gonzalo *et al* [14].

Taken together, these results suggest that the i.n. administration of ACh-DC promotes profound structural changes in the lung parenchyma during the development of a short-term model of OVA-induced lung inflammation, some of these characteristic of inflammatory chronic stage [34].

Several reports have shown that lung damage and dysfunction are associated with inflammatory infiltrate and mediators produced by it, during allergic inflammation [34]. Moreover, besides macrophage and DC activity in lungs as antigen-presenting cells, they may lead, directly or indirectly, to the secretion of cytokines that initiate phenotypic changes in the airway epithelium [14,34]. In agreement with this, we have shown here that ACh-treated DC produced a higher amount of acute mediators of inflammation, such as TNF-α and CCL2/MCP-1, associated with some of the initial characteristics of inflammatory processes: increased vascular permeability, monocyte recruitment and tissue damage [14,35]. Moreover, TNF-α is exacerbated in airways of asthmatic patients and contributes to the development of airway remodeling [35]. Also, it was demonstrated that this cytokine can reach a high serum level in patients with severe asthma and it has been suggested as marker of “systemic” inflammation [36,37]. Related to this, some experimental studies in asthma models have reported that some agents inhibiting the synthesis and activity of TNF-α as well as blocking agents of this cytokine decreased/suppressed the airway inflammation and improved the lung histopathology [35].

These above-mentioned reports are consistent with our results shown here; we found a higher TNF-α *in vivo* production at the end of the model (14 days after instillation of DC) in mice instillated with ACh-treated DC respect to mice instillated with untreated DC (Fig 7D), supports the impact of cholinergic activation of DC on the onset of airway inflammatory response, which is capable of exacerbating it and promoting lung injury by the induction of mononuclear cell recruitment. Our results could contribute to better understanding the onset of the inflammatory response that leads to the increase of TNF-α observed in airways and sera of asthmatic patients.

In agreement with our results, Gonzalo *et al* showed that monocytes/macrophages are the first cells accumulated at early stages of OVA-induced lung inflammation [13,14,33], and that their recruitment is mainly dependent on the local production of CCL-2/MCP-1 in lungs [13,14]. Interestingly, the neutralization of this chemokine at the beginning of the inflammatory process has been showed to reduce bronchial hyperresponsiveness associated to a strongly decrease not only in monocytes/macrophages but also in lymphocytes and eosinophils recruitment [14]. These findings support the key role of CCL-2/MCP-1 acting up-stream of the inflammatory response.

There is no doubt about the relevance of the neuronal cholinergic system in the regulation of airways [38]. It is known that the parasympathetic neuronal network penetrates deep into the airway wall, affecting the bronchoconstriction and the mucus release from submucosal glands, and in lesser degree, from goblet cells in the airway epithelium [39]. However, neuronal system is not the only source of ACh in the airways. The nonneuronal ACh synthesis system has been detected on various cells types, such as epithelial, fibroblasts and inflammatory cells [5,38].

In lungs, the synthesis and the release of non-neuronal ACh occur in inflammatory processes [40–43] as well as in homeostatic regulation [44], stimulating cholinergic receptors in both autocrine or paracrine manner [38]. Moreover, different immune cells such as macrophages, B and T lymphocytes [7] and even DC [6,16] express choline acetyltransferase or ChAT, the enzyme responsible of ACh synthesis. The ChAT expression in all of them was confirmed with the recently developed Fluorescent ChAT-Reporter Protein transgenic mice [4,45–48]. Particularly, Koarai et al. [21] reported the expression of ChAT mRNA in human lung and alveolar macrophages and monocytes.

Taking this background account, we suppose that at the beginning of lung inflammatory disease, the ACh released by airway epithelial and resident immune cells could be relevant in the DC activation and moreover, the production of ACh by recruited immune cells might contribute to the inflammatory response perpetuation.

## Conclusion

Our results suggest that ACh promotes the acquisition of an inflammatory profile on murine DC through mAChR, which is characterized by an enhanced expression of MHC II as well as an increased production of TNF-α and MCP-1, both strongly associated with inflammatory infiltrate and tissue damage in several pathologies. Interestingly, our observations made in a short-term experimental model of OVA-induced lung inflammation reveal that the early instillation of ACh-treated DC promotes increased lung injury that reaches systemic levels of inflammation compared to untreated DC, suggesting a potential role for ACh in the modulation of immune mechanisms underlying pulmonary inflammatory process.

## Supporting information

S1 Fig TNF-α production of maturated bone marrow-derived DC. DC (1x10^6^ cells/ml) were incubated with or without 1μg/ml LPS for 18 h. TNF-α secretion was measured by ELISA (mean ± SEM, n = 4 experiments). *p<0.05 Wilcoxon test.
(TIF)

S2 Fig ACh-DC not modulates CD4^+^, CD8^+^ and B220^+^ cell population in OVA-induced lung inflammation. Cells isolated from lungs were stained with mAb directed to CD4 and CD8 (**A**) or B220 (**B**) and analyzed by flow cytometry, gating mononuclear cells and excluding for the analysis the High Autofluorescence Cells. Representative experiments are shown (N = 2).
(TIF)
