# Supplementary figures and images for: Acetylcholine-treated murine dendritic cells promote inflammatory lung injury
Source: PLoS One. 2019 Mar 1;14(3):e0212911. doi: 10.1371/journal.pone.0212911 (PMC6396899; doi:10.1371/journal.pone.0212911)

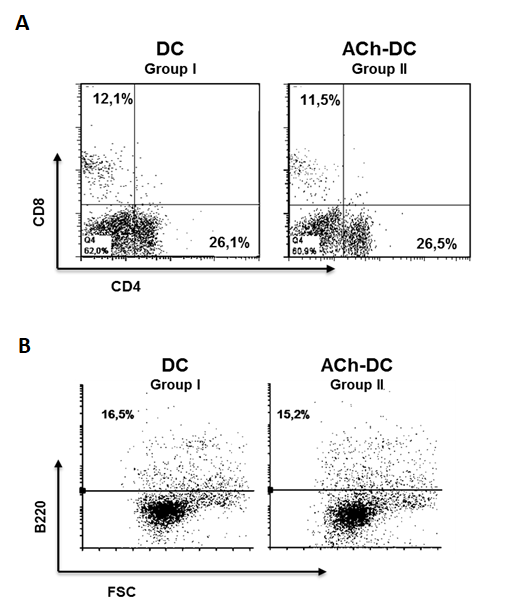

Supplement: S2 Fig — Cells isolated from lungs were stained with mAb directed to CD4 and CD8 (A) or B220 (B) and analyzed by flow cytometry, gating mononuclear cells and excluding for the analysis the High Autofluorescence Cells. Representative experiments are shown (N = 2). (TIF) [file pone.0212911.s002.tif]
